# Role of Host-Mediated Post-Translational Modifications (PTMs) in RNA Virus Pathogenesis

**DOI:** 10.3390/ijms22010323

**Published:** 2020-12-30

**Authors:** Ramesh Kumar, Divya Mehta, Nimisha Mishra, Debasis Nayak, Sujatha Sunil

**Affiliations:** 1 Vector Borne Disease Group, International Centre for Genetic Engineering and Biotechnology, New Delhi 110067, India; kumar.ramesh475@gmail.com (R.K.); divyamehta2975@gmail.com (D.M.); nimisha.mishra17@gmail.com (N.M.); 2 Department of Biosciences and Biomedical Engineering, Indian Institute of Technology, Indore 453552, India; nayakdn@iiti.ac.in

**Keywords:** RNA viruses, post-translation modification, pathogenesis, ubiquitination, acetylation, glycosylation, phosphorylation, host factors, virulence factors

## Abstract

Being opportunistic intracellular pathogens, viruses are dependent on the host for their replication. They hijack host cellular machinery for their replication and survival by targeting crucial cellular physiological pathways, including transcription, translation, immune pathways, and apoptosis. Immediately after translation, the host and viral proteins undergo a process called post-translational modification (PTM). PTMs of proteins involves the attachment of small proteins, carbohydrates/lipids, or chemical groups to the proteins and are crucial for the proteins’ functioning. During viral infection, host proteins utilize PTMs to control the virus replication, using strategies like activating immune response pathways, inhibiting viral protein synthesis, and ultimately eliminating the virus from the host. PTM of viral proteins increases solubility, enhances antigenicity and virulence properties. However, RNA viruses are devoid of enzymes capable of introducing PTMs to their proteins. Hence, they utilize the host PTM machinery to promote their survival. Proteins from viruses belonging to the family: *Togaviridae*, *Flaviviridae*, *Retroviridae,* and *Coronaviridae* such as chikungunya, dengue, zika, HIV, and coronavirus are a few that are well-known to be modified. This review discusses various host and virus-mediated PTMs that play a role in the outcome during the infection.

## 1. Introduction

Viruses are intracellular pathogens that infect a wide range of organisms, from single-cell bacteria to multicellular organisms like plants and animals. Most of the RNA viruses have a small genome (typically <14 kb) [1], while coronavirus, toroviruses, and roniviruses have larger genome sizes (24–30 kb) [1,2]. Regardless of the genome sizes, viruses depend on the host cell machinery for various biological and physiological functions associated with their genome replication, transcription, translation, packaging, and release from infected cells.

**Figure 1:**
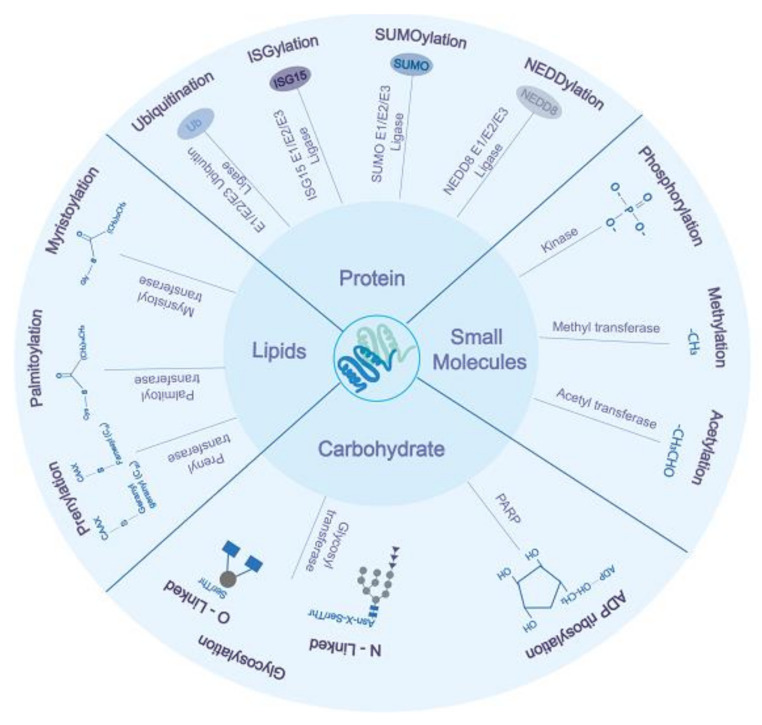
Overview of various post-translational modifications of virus and host proteins having an important role during viral infections. Based on the biochemical nature of attached moieties, post-translational modification (PTMs) can be categorized into modification by small protein groups (ubiquitination, ISGylation, SUMOylation, NEDDylation), carbohydrates moieties (glycosylation, ADP ribosylation), lipids (palmitoylation, myristoylation, and prenylation), and small chemical groups (phosphorylation, methylation, and acetylation). The proteins upon modification result in loss/gain in function, which modulates the virus’s life cycle and host response to virus infection.

Post-translational modifications (PTMs) of proteins are essential for cellular housekeeping functions of the cells. PTMs include adding small proteins or functional groups such as ubiquitination, lipidation, glycosylation, methylation, phosphorylation, and acetylation to specific amino acids within the protein [3,4,5]. PTMs are carried out by specialized enzymes, such as ubiquitin E3 ligase, glycosyltransferase, poly ADP (Adenosine diphosphate) ribose polymerase (PARP), acetyltransferase, and kinases [3,6]. PTMs enhance protein solubilization, conformation (by altering charge or hydrophobicity), interactions, signaling, degradation, and thus play a crucial role in cell growth [7]. During infection, PTMs promote the virus growth by enhancing viral replication, assembly, release, and interferon response inhibition. In contrast, the host overcomes viral infection by inactivating viral proteins by either removing PTMs, which are crucial to the enzymatic activity of viral proteins or attaching small molecules such as ubiquitin or ubiquitin-like proteins leading to their inactivation and/or proteasomal mediated degradation. This review discusses some essential PTMs associated with host-pathogen interplay related to RNA viruses. Based on the size/chemical nature, PTMs can be broadly categorized into four categories: (1) Protein-based modification involving ubiquitin (known as ubiquitination), SUMO (Small ubiquitin-like modifier, known as SUMOylation), ISG (Interferon-stimulated gene, known as ISGylation), NEDD8 (Neural precursor cell expressed, developmentally down-regulated 8, known as NEDDylation), (2) carbohydrate molecule-based modification such as glycosylation, ADP ribosylation, (3) lipid molecule-based modifications such as palmitoylation, myristoylation, prenylation, and (4) chemical/ionic group such acetyl, phosphate, and methyl to the nascent proteins (Figure 1).

## 2. Protein-Based PTMs

**Figure 2:**
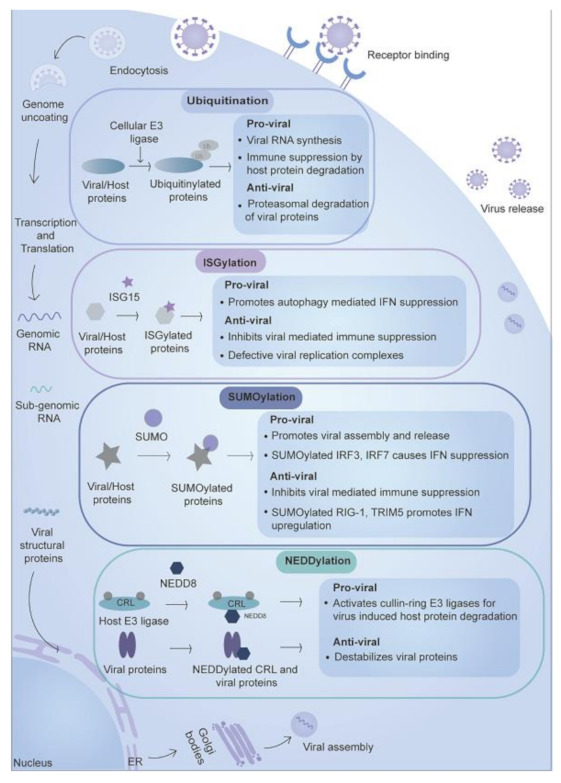
Small protein-based post-translational modifications during viral infection. Ubiquitin and ubiquitin-like molecules such as ISG15, NEDD8, and SUMO play an essential role during viral infection. Specific enzymes such as E1, E2, and E3 ligases are involved in the modification process. The anti-viral effect is exerted by inducing IFN response, destabilizing viral proteins, or targeting viral proteins to their degradation. These modifications also exert a pro-viral impact by improving the enzymatic activity or interactions of the viral proteins, which promotes viral replication and degradation of host proteins involved in immune response proteins.

Proteins such as ubiquitin and ubiquitin-like proteins (Ubls) including SUMO (Small ubiquitin-like modifier), NEDD8 (Neuronal precursor cell-expressed developmentally down-regulated protein 8), ISG15 (Interferon-stimulated gene 15), FAT10 (Human leukocyte antigen F locus adjacent transcript 10), URM1(Ubiquitin-related modifier-1), UFM1 (Ubiquitin-fold modifier 1) and ATG8 (Autophagy-related protein 8) are attached to the host and viral proteins involving activating enzyme E1, ubiquitin-conjugating enzyme E2 and ubiquitin ligase E3 [8]. E1 activates the ubiquitin or ubiquitin-like proteins and then transfers them to E2, which then transfers the molecule to the protein substrate via the activity of E3 ligase. These small proteins have a similar 3D structure like that of ubiquitin (β1 β2 α1 β3 β4 α2 β5), but lack sequence similarity and are called ubiquitin-like proteins [9]. The size of these PTMs varies from 8 kDa for ubiquitin and 17 kDa for ISG15, as they have other peptide chains in addition to the ubiquitin-like domain. Among the ubiquitin-like protein modification, the role is well-known in ubiquitin, SUMO, NEDD8, and ISG15 during the viral infection, while for others such as, FAT10, URM1, UFM1, and ATG, very few reports are available. These modifications act as anti-viral by either activating immune response or targeting the viral proteins to degradation or act as pro-viral by downregulating immune response or activating enzymatic/functional activities of viral proteins (Figure 2). We describe below the various ubiquitin-like modifications and their role during virus infection.

### 2.1. Ubiquitination 

Ubiquitination is a vital modification that plays a role in various processes and maintains homeostasis under normal physiological conditions and disease/infections [10]. It involves covalent attachment of ubiquitin protein (of around 76 amino acids long) to C-terminal glycine residue of target protein via lysine amino acid of ubiquitin (Ub) known as the canonical pathway. The other pathway, called a non-canonical pathway, involves cysteine, serine, and threonine to a target protein [11,12]. These modifications play both pro-viral and anti-viral roles during viral infections and are described below.

#### 2.1.1. Role of Ubiquitination in the Inhibition of Viral Replication 

As an innate mechanism to counter viral infection, host performs ubiquitination of viral proteins and targets them for degradation, limiting the viral spread (Figure 2) [13,14]. Various ubiquitin ligases such as TRIM5a (Tripartite motif-containing 5a) [15], TRIM24 [16], TRIM33 [17], TRIM41 [18], and E6AP, also identified as Ubiquitin-protein ligase (E3A) [19], are known to ubiquitinate viral proteins. Ubiquitination during viral infection negatively affects the viral replication by:

(i) Tagging protein with Ub and targeting them to proteasomal degradation, e.g., ubiquitination of Dengue (DENV) NS3 results in its proteolytic degradation [13]. DENV NS3 forms a protease complex (NS2B3) with NS2B and cleaves cGAS (cyclic GMP-AMP synthase) and STING (stimulator of interferon genes) [20,21]. Thus, degradation of NS3 negatively affects viral replication by inhibiting the protease complex formation (which is needed for viral protein processing) and increasing the interferon response due to less degradation of STING and cGAS. Nucleoprotein (NP) of Influenza, a virus (IAV), and vesicular stomatitis virus (VSV) are targeted for ubiquitin-mediated proteasomal degradation. As NP is a structural component of the negative-sense RNA virus genome, a low level of nucleoprotein allows host machinery to degrade viral RNAs, thereby limiting viral infection [18,22]. Other examples include HIV (Human immunodeficiency virus) integrase (IN) protein, which is polyubiquitinated and targeted to proteasomal degradation and affects viral replication as well as pro-viral DNA formation [17,19]. HCV (Hepatitis C virus) core protein, which is also ubiquitinated and targeted to degradation [19] thus negatively affecting the crucial viral processes such as replication, assembly as well as a counter-response to the interferon-mediated attack on the virus (Figure 2).

(ii) Inhibition of complex viral formation leading to a reduction in viral replication. DENV NS1 forms a replication complex with NS4B (non-structural 4B). Its ubiquitination prevents its association with NS4B (non-structural 4B) and promotes its oligomerization, thus preventing replication complex formation [23], leading to restricted viral replication. Besides ubiquitinating viral proteins, E3 ligases auto ubiquitinate various TRIM (Tripartite motif) proteins. A few examples are TRIM5a [14] and TRIM26 [24], which lead to enhanced expression of interferons or degradation of viral proteins, thus protecting the host. A few more examples are also shown in Table 1, which include host-mediated ubiquitination and deubiquitination of the host and viral proteins to inhibit viral replication, to give brief information about the different strategies used by the host to counter viral growth via ubiquitin-mediated post-translation pathway.

#### 2.1.2. Role of Ubiquitination in the Promotion of Viral Replication

The ubiquitination of viral proteins in some cases promotes the viral replication through various mechanisms such as (i) ubiquitin-dependent enzymatic activity of viral proteins, e.g., Ebola virus protein VP35, which is a cofactor of the viral polymerase as well as an inhibitor of the host anti-viral type I interferon (IFN-I) system [6], PB2 (Polymerase basic protein 2) (which enhances polymerase action and thus viral replication), nucleoprotein of influenza virus (promote replication and assembly) [25,26,27], and HCV NS2 (which promotes viral assembly) [28]. Ubiquitination of the polymerase subunit of these viruses positively affects enzymatic activity, thus promoting viral replication (Figure 2).

(ii) Ubiquitin-mediated negative effect on the host immune response leading to a positive effect on viral replication, e.g., Zika virus (ZIKV) NS1 interacts with USP8 (Ubiquitin Specific Peptidase 8) and causes the deubiquitination of Caspase 1 and prevents its proteasomal degradation. Enhanced stabilization of Caspase 1 promotes the cleavage of cGAS, which is involved in the initiation of IFN-I signaling [43]. NS5 of ZIKV and yellow fever viruses bind to and prevent the ubiquitination of RIG-1 (Retinoic acid-inducible gene 1), followed by inhibition of both phosphorylation and nuclear translocation of IRF3 (IFN regulatory factor 3) [44], leading to the suppression of IFN response. ZIKV NS3 and NS2B3 bind to host MAVS (Mitochondrial anti-viral signaling protein) and MITA (Mediator of IFN regulatory factor 3 activation) and target them for degradation by catalyzing their K48-linked ubiquitination [45].

(iii) Host and viral encoded deubiquitinating enzymes remove ubiquitin and promote viral growth. Viruses negatively affect innate immune response by deubiquitinating host immune pathways proteins [46] e.g., SARS-CoV (Severe acute respiratory syndrome coronavirus) and MERS-CoV (Middle east respiratory syndrome coronavirus) encoded protein such as PLpro (papain-like protease) exhibit deubiquitinating enzymatic activity and deISGylating activity and affect cytokine and interferon response [47,48] by reducing the expression of CCL5 (Chemokine ligand 5), CXCL10, (C-X-C motif chemokine ligand 10) and IFNβ [49]. MERS-PLpro displays broad cleavage specificity towards poly-Ub chains and suppresses the IFNβ signaling pathway [50]. SARS-PLpro cleaves K48 (preferentially) and K63 -linked poly-Ub chains of Tumor necrosis factor receptor (TNFR)-associated factor 3 (TRAF3) and TRAF6, thereby inhibiting NF-kB (Nuclear factor kappa B) activation [51], whereas SARS-CoV-2-PLpro have a preference for ISG15 as compared to ubiquitin [48,52]. Additionally, host deubiquitinating enzymes USP7 promotes HIV replication by removing ubiquitin from Tat (Trans-Activator of Transcription) protein, which enhances the viral transcription efficiency, rescuing it from degradation [53]. These examples show that both ubi and deubiquitination play an important role in regulating immune response and viral growth.

### 2.2. ISGylation 

Viral infections induce an immune response, an anti-viral state in cells by inducing expression of IFN-I production downstream of the JAK-STAT (Janus kinase-signal transducer and activator of transcription) pathway leading to increased transcription of ISGs (Interferon-stimulated genes) [54]. Microarray studies suggest around 300 ISGs are involved in various anti-viral defense functions. These include but are not limited to cellular signaling, inflammation, transcription activation, antigen processing, and immune modulation [55]. Among the ISGs, ISG15 plays a role in PTM. In contrast, GBP1 (Guanylate-binding protein 1), IFIT1 (Interferon Induced Protein With Tetratricopeptide Repeats 1), IFIT2, ISG20, ZAP (zinc finger anti-viral protein) bind to viral RNA or inhibit apoptosis [56]. Here we will discuss the anti-viral and pro-viral actions of ISG15 as PTM, as shown in Figure 2 and Table 1.

#### 2.2.1. Role of ISGylation in the Inhibition of Viral Replication

ISG15 conjugation plays an anti-viral role during viral infection by conjugation to viral proteins or hosts cellular proteins, leading to inhibition of enzymatic activity, viral replication inhibition, translational shutoff, and metabolic reprogramming [57,58]. ISGylation inhibits the viral cycle by (i) targeting viral replication and release. ISG15 interferes with Ebola VP40 (matrix protein) mediated viral egress. It targets and negatively affects Nedd4 ligase, which ubiquitinate VP40 and is helpful for the VP40 activity. ISGylation leads to a reduction in the viral release [57]. Influenza B virus (IBV) viral genome occurs in the form of ribo-nucleoprotein complex (RNP). The RNP assemblies facilitate IBV RNA synthesis and comprise a viral polymerase, viral RNA, and NP oligomers. During viral infection, ISGylation of viral NP blocks viral RNA synthesis by inhibiting oligomerization leading to reduction of viral RNA synthesis. NS1B reduces modified NP by sequestering to minimize the detrimental effect ISGylation; however, dominant-negative effect (a minute fraction of modified NP left from NS1B sequestration) negatively affects viral RNA synthesis [59] (Figure 2).

(ii) Enhancing the interferon response. ISGylation induced anti-viral in non-hematopoietic cells during Coxsackievirus B3 (CVB3) infection. The authors suggest that ISG conjugation to anti-viral effectors IFIT1 and IFIT3 blocks ubiquitination-dependent degradation and stabilizes these proteins leading to an enhanced anti-viral response (Figure 2) [60]. CVB3 encodes a protease (2APro) that mediates host cell translational shut off by cleavage of eukaryotic translation factor 4γ1 (eIF4G1), which binds to RNA cap involved in cap-dependent translation. The eIF4G1 degradation favors the viral RNA translation as the CVB3 genome lacks cap and is translated in a cap independent manner. However, ISGylation of 2APro impairs the eIF4G1 cleavage during CVB3 infection, causing suppression of viral replication [4].

#### 2.2.2. Role of ISGylation in Promotion of Viral Replication

ISGylation is a part of the anti-viral defense system, which helps in controlling viral replication. In some cases, the modification serves the pro-viral function for the virus via inhibition of the immune pathways. During RNA virus infection, LRRC25 (Leucine-rich repeat-containing protein 25) interacts with ISG15 modified RIG-1 and promotes degradation via autophagy in a p62-dependent manner, thereby downregulating IFN response (Figure 2) [61]. Studies with SARS-CoV2-PLpro found that it blocks the IRF3 pathway (by inhibiting the phosphorylation of IRF3) and NF-kB pathway (by an unknown mechanism) [52,62].

### 2.3. SUMOylation 

SUMOylation requires conjugation of a small ubiquitin-like modifier (SUMO) to a lysine residue in the consensus sequence ΨKxD/E within the target protein (Ψ represents hydrophobic amino acid residue, x is any amino acid, D/E is an acidic amino acid). Proteins can be mono- or poly-sumoylated [63]. SUMOylation regulates critical cellular processes such as transcriptional regulation, DNA repair, and innate immunity [9]. Covalently attached SUMO moieties regulate protein stability, localization, and interactions by bringing about conformational changes in target proteins [64]. In addition to covalently linked SUMO chains, proteins with a SUMO-interaction motif (SIM) can interact with SUMO moiety non-covalently. Such non-covalent interactions assist in ubiquitination due to the SIM motif in SUMO-targeted ubiquitin ligases (STUbLs) [65]. SUMOylation can inhibit viral replication by promoting immune response and act as a pro-viral by tagging host proteins, which hampers IFN response (Figure 2). The outcomes of modifications are explained below and Table 1.

#### 2.3.1. Role of SUMOylation in the Inhibition of Viral Replication 

Similar to ubiquitination, SUMOylation can inhibit viral replication. SUMO conjugation to virally encoded proteins is implicated in stabilization and pertains to later viral assembly stages and release. SUMOylation of HIV-1 p6 (gag proteolytic product) has a negative impact during early viral replication [66]. SUMOylation limits another HIV-1 protein, integrase (IN). Mutations in the phylogenetically conserved SCMs (SUMOylation consensus motifs) of HIV-1 IN protein results in the reduction of infectivity, replication kinetics, and integration events [67]. In addition to SUMOylating the viral proteins, host proteins belonging to pathways such as cell cycle, mRNA processing, transcription elongation, and immune pathways are often SUMO-modified [68]. The earlier investigation has shown SUMOylation of RIG-1 during RNA virus infection promotes its ubiquitination, leading to increased interaction with Cardiff, a mitochondrial adaptor in RIG-1 anti-viral pathway resulting in enhanced IFN signaling, which affects viral replication [69].

#### 2.3.2. Role of SUMOylation in the Promotion of Viral Replication 

Contrary to the SUMO modifications mentioned earlier in viral proteins, which leads to inhibition of viral replication, SUMOylation modification of proteins is also involved in enhancing viral replication e.g., ebola virus VP35 protein promotes the SUMOylation of host interferon response components (IRF3 and IRF7) via SUMO E3 ligase PIAS1, significantly hampering IFN promoter activity (Figure 2). [70]. Sumoylation of SARS-CoV NP promotes viral RNP formation and nucleocapsid assembly, which favor viral replication and replication [71]. SUMOylation of IAV matrix protein (M1) and NP proteins results in the efficient assembly of viral RNP-M1 complexes and consequent viral maturation and release [72,73]. In addition to this, SUMOylation inhibits IFN response in various viruses that promote viral growth, e.g., Ebola virus VP24 protein upon sumoylation binds to USP7 and is deubiquitinated. It binds to karyopherin alpha1, a STAT1 NLS (Nuclear localization signal) receptor, and inhibits IFN response [74,75], thus favoring viral replication. SUMOylated ZIKV NS5 binds to STAT1 and prevents its association with PML (promyelocytic leukemia), which is needed for ISG expression leading to reduced ISG expression [76].

### 2.4. NEDDylation

NEDDylation is a process where target proteins undergo covalent attachment to ubiquitin-like molecule NEDD8 (neural precursor cell expressed, developmentally down-regulated 8) [77]. NEDDylated substrates have been implicated in cancer, DNA damage responses, transcriptional regulation, nucleolar stress signaling, apoptosis, and ubiquitin-proteasome machinery [9]. NEDDylation, an essential regulator of CRL (Cullin-RING ubiquitin ligase) activation, is an attractive target for viruses to hijack host ubiquitin machinery. By doing so, viruses modulate myriads of cellular pathways enhancing viral survival and growth in the host [78].

Although some viral proteins have also been identified as targets of neddylation, most studies focus on ubiquitin machinery’s viral intervention via neddylation. Modulation in the neddylation pathway has been studied in very few representative viruses such as influenza and HIV [5,79]. For the context of this review, we will focus mainly on the intervention of RNA viruses in host neddylation machinery and vice versa.

#### 2.4.1. Role of NEDDylation in the Inhibition of Viral Replication 

NEDDylation, like other ubiquitin-like modifiers, is known to target the viral proteins to degradation (Figure 2) [5]. Upon viral entry into the cell, multiple anti-viral cascades are triggered. One of them involves phosphorylation and homodimerization of IRF3, leading to its activation. Activated IRF-3 is translocated to the nucleus, where it acts as a transcription factor for multiple anti-viral genes, including IFN-I, thus mounting cellular defenses [80]. To counteract the IFN response, viruses cause degradation of IRF-3 by hijacking RING-ligases. For example, the Sendai virus (SeV) upon infection causes NEDDylation-dependent proteasomal degradation of IRF3 by utilizing Cul1 (Cullin-1) based ubiquitin ligases [81]. HDM2 E3 ligases target IAV PB2 (Polymerase basic protein 2) for NEDDlation (K699). Conjugation of NEDD8 to IAV PB2 harm the protein stability and viral replication. In contrast, the mutant (K699R) showed heightened virulence during infection in mice, highlighting the importance of NEDDylation on viral proteins and replication [5]. IRF3 and IRF7 were also identified as targets for NEDD8 conjugation [77], suggesting a direct link between the neddylation of viral proteins and viral replication inhibition. Moreover, the study established that NEDD8 deficient zebrafish larvae and adults were more susceptible to SVCV (Spring viremia of carp virus) infection than wild type [77]. 

#### 2.4.2. Role of NEDDylation in the Promotion of Viral Replication

NEDDylation of proteins leads to their degradation, and this has a pro-viral effect when the modification occurs on the immune pathway proteins. During viral infection, this hampers the immune pathway and promotes viral replication. The NEDDylation of CRL4 and CRL5 causes ubiquitin-mediated proteasomal degradation of APOBEC3G (A3G) (which belongs to the family of cytidine deaminases and restricts retroviral replication to protect the infected host by inducing hypermutations) and SAMHD1 (a deoxynucleoside triphosphohydrolase that blocks retrovirus infection at reverse transcription). It promotes viral replication (Figure 2) [79,82]. NEDDylation of Cullin-1 during Influenza virus infection causes its inhibition and negatively affects the NF-kB pathway. Inhibition of the neddylation pathway by targeting Nedd8-activating enzyme subunit 1 (NAE1) with inhibitor (MLN4924) suppresses virus replication [83], suggesting the important role of NEDD8 in viral replication. 

## 3. Carbohydrate-Based Post-Translational Modifications

Carbohydrate moieties as mono/poly and homo/heteropolymers are attached to the proteins. The transfer of moieties is facilitated by a special class of enzymes called glycosyltransferases [84]. These modifications serve the purpose of solubilization, receptor-ligand binding, which is crucial during viral protein function, viral complex assembly, viral entry, host-pathogen interactions, viral protein secretion, virulence, and antigenicity [85]. Beside eukaryotes, where it is well known to play a role in cells’ function, it also plays a vital role in the life cycle of viruses such as HIV, flavivirus, alphavirus, influenza, and coronavirus [86]. The envelope and secreted viral proteins are modified with carbohydrate moieties, where these moieties assist in protein folding and assembly. Glycosylation of secreted viral proteins often subverts humoral immune response [86,87]. 

### 3.1. Glycosylation

**Figure 3:**
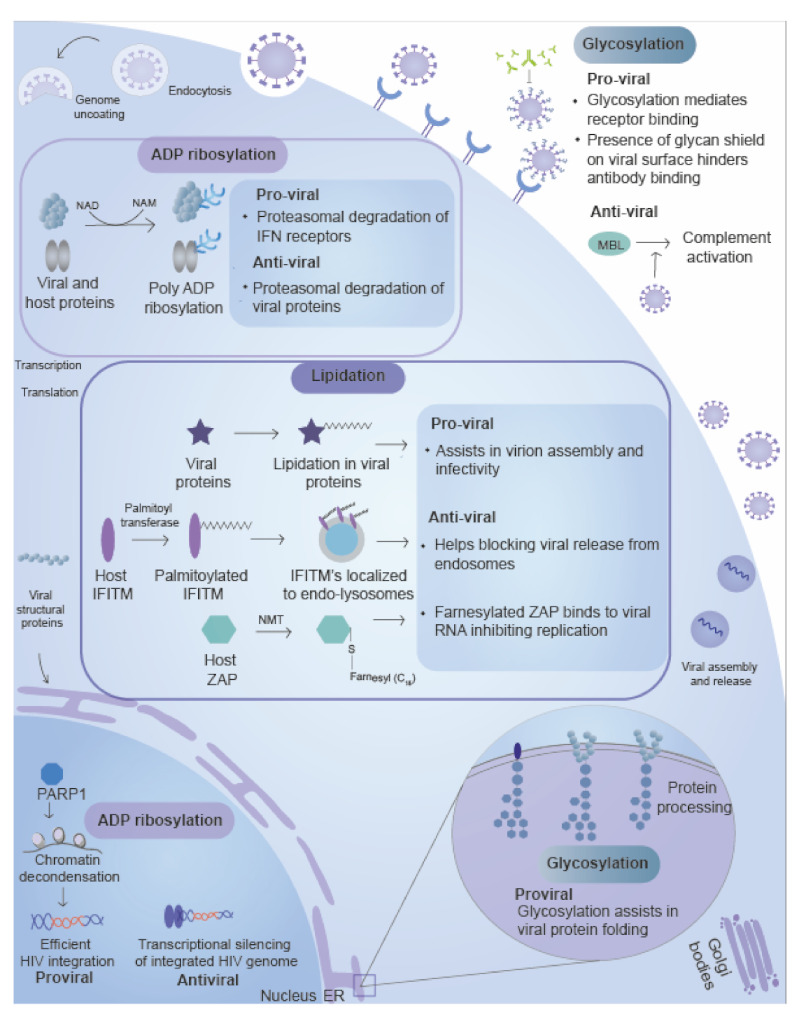
Carbohydrate and lipid moieties based post-translational modification. Carbohydrates based PTMs includes glycosylation and ADP ribosylation. Glycosylation plays a role in enhancing viral replication by increasing receptor binding, protein solubilization, virulence, and antigenicity of viral proteins. Glycosylation is also involved in the inhibition of viral replication. Mannose-binding lectin (MBL) binds to non-self glycoproteins such as viral proteins, activates the complement pathway, and inhibits viral replication. PARPs (Poly ADP ribose polymerases) mediated ADP ribosylation of viral proteins targets them for degradation, thus acting as anti-viral. The modification also works as pro-viral by targeting protein involved in the immune response during viral infection. Viruses also favors their growth by removing the ADP ribosylation, thereby favoring growth. Lipid molecules such as palmitoylation, myristoylation, and prenylation. The modifications favors viral replication by assisting in viral assembly. In contrast, these inhibit viral replication by triggering IFN response, bind to viral RNA, and inhibit viral replication or block membrane fusion.

Glycoconjugates are macromolecules composed of carbohydrate chains (made up of homo or heteropolymers of either fucose, mannose, sialic acid, N-acetylglucosamine (GlcNAc), or Galactose) covalently attached to the protein in a process termed as glycosylation [88,89]. An array of the glycoconjugates exist within the intracellular and extracellular milieu, and these differ in the glycan composition, anomeric ring linkages, length, and site of occurrence. Glycosylation of proteins is classified into mainly two types: N-linked glycosylation, where the glycan moiety is linked to asparagine, and O-Linked glycosylation, where the glycan moiety is attached to oxygen atom present on the hydroxyl group of serine or threonine amino acid residue within a protein [88]. In addition to playing an essential role in cells’ functioning, glycosylation plays a significant role in the life cycle of RNA viruses, including from genus *Alphavirus*, *Flavivirus*, *Ebolavirus*, *Betacoronavirus*, *Arenavirus,* and *Henipavirus* [85]. The glycan moieties play a crucial role in viral entry, assembly, virulence, pathogenicity (Figure 3) and are discussed below.

#### 3.1.1. Role of Glycosylation in the Inhibition of Viral Replication

Glycosylation help viruses at various stages of their life cycle. It also favors the host by recognizing viral proteins, activating the immune system. Viral proteins, besides helping in virus replication, assembly are internalized by immune cells (macrophages and dendritic cells) and result in MHC (Major histocompatibility complex) mediated antigen presentation and virus epitope-specific antibody generation [90,91]. The antibodies are glycosylated at Fc (fragment crystallizable) region before their secretion. Glycosylation helps in their solubilization and complement activation. Their glycosylation pattern changes among different viruses such as HIV-1, Influenza, DENV, and Ebola and has different avidity and affinity [92]. Glycosylation affects humoral response via the complement system recruitment [93], which leads to restriction in viral replication. Secreted antibodies play a role in restricting viral infection by virus neutralization, complement activation, and antibody-dependent cellular phagocytosis (ADCP). Antibodies against viral proteins such as HCV E2 protein restrict viral infection by binding to viral proteins involved in assembly or pathogenicity [94], thereby restricting viral replication. Antibody binding to viruses/viral proteins leads to the formation of complexes that are recognized by immune cells leading to their phagocytosis mediated clearance. This phenomenon is observed in HIV-1 and influenza, however, there are few exceptions like ZIKV, DENV, yellow fever virus where this leads to enhancement in viral replication due to antibody-dependent enhancement (ADE) [95,96,97], a phenomenon of phagocytosis of virus antibody complex which has higher efficiency of entry but escapes phagocytic degradation due to non-neutralizing antibodies which lead to viral escape. In addition to antibodies, Mannose-binding lectin (MBL) also restricts viral infection. MBLs are pattern binding proteins that differentiate self from non-self protein based on their glycosylation pattern with their carbohydrate recognition domains (CRDs). These are known to inhibit viral (SARS-CoV and DENV) replication by activating a lectin-based complement system (Figure 3) [98,99].

#### 3.1.2. Role of Glycosylation in the Promotion of Viral Replication

Viruses, being opportunistic pathogens, depend on the host glycosylation machinery for glycan conjugation on their proteins [87]. Glycosylation is involved at various stages of viral replication to promote its amplification. These involve (i) Receptor binding of the virus inside the cell. Glycosylation assists viruses in receptor binding and subsequent infection of immune cells. DC-SIGN (Dendritic cells-soluble extracellular dendritic cell-specific ICAM3 grabbing non-integrin) receptors present on the surface of immune cells such as dendritic cells and macrophages facilitate viruses such as alphavirus (SINV) and flavivirus (DENV) entry by interacting with glycans frequently present on the viral envelope proteins (Figure 3) [100,101].

The Ebola virus attachment and entry into cells is mediated by surface GP1 (glycoprotein subunit 1). The GP1 subunit mediates receptor binding and is enriched by O-linked glycan moieties [102]. The glycan moieties shield antigenic epitopes on the GP1 itself and impair antigenic presentation by sterically hindering accessibility to host surface proteins such as MHV-I and integrins, thus severely impending host immune response (Figure 3) [103]. Like Ebola, viral attachment glycoprotein (G) encoded by the RSV (Respiratory syncytial virus) has high glycosylated O-glycans and has role in the fusion of virus and cell membrane [104].

(ii) Viral replication and maturation. Glycosylation of viral envelop proteins is the most well- known example of glycosylation in the virus. The modification helps the proteins to adopt confirmation and ultimately help in replication as well as maturation. A few examples are: envelop protein of West Nile virus (WNV), Influenza, HIV-1, and ZIKV [105,106,107]. The importance of the glycosylation on envelop proteins can be understood from the fact that inhibition of glycosylation severely affects the maturation process in viruses such as Ebola, Influenza, HIV-1, and ZIKV [106,107]. 

(iii) Viral pathology. Glycans also play a crucial role in pathology during viral infection. Glycosylation promotes pathogenesis by enhancing the binding of viral proteins to cells leading to enhanced infection. N glycan moieties on envelop protein in ZIKV, Influenza, HIV-1, Ross River virus (RRV) promote virulence [107,108,109]. In addition to glycosylation of structural proteins, non-structural proteins such as DENV NS1 are glycosylated and have role in protein secretion and stability of hexameric form which help in immune evasion by binding to lectin pathway proteins such as C1s, C4, C4-binding protein, MBL and prevent lectin complement activation and DENV neutralization, thus regulate pathogenesis [110,111].

(iv) Immune evasion. Glycosylation helps the virus to escape the immune system by avoiding recognition. Some viruses display heavily glycosylated surface envelope proteins to escape humoral immune recognition. HIV-1 envelope is a trimer of non-covalent gp120-gp41 dimers. These subunits contain numerous glycan moieties with a gp120 monomer alone constituting 18–33 glycans (oligo-mannose type) and act as a shield against the immune system [112]. The glycan moieties are weakly immunogenic by themselves, and glycan shield sterically hinders neutralizing antibodies from penetrating and binding to epitopes on the underlying envelope protein [113]. However, glycan holes, which represent areas close to the inter-protomer axis, the CD4-binding site, and the fusion peptide, are accessible to neutralizing antibodies [114].

### 3.2. ADP-Ribosylation

ADP-ribosylation is a ubiquitous modification present across all life domains ranging from the virus to eukaryotic organisms [115,116,117]. ADP-ribosylation utilizes nicotinamide adenine dinucleotide (NAD) as a cofactor to transfer ADP-ribose nucleotide onto proteins and DNA [115]. The reaction is catalyzed by the enzyme ADP-ribosyl transferases known as ARTDs (ADP-ribosyltransferase, diphtheria toxin-like) or PARPs (Poly ADP ribose polymerase) [115]. The ADP-ribosylation plays an essential role in multiple biological processes such as viral replication, translation, signal transduction, epigenetics, cellular stress response, and protein degradation [118]. Modification by PARPs occurs mostly on acidic amino acid residues such as glutamate and aspartate, but other residues such as serine, arginine, lysine, and cysteines can also act as acceptors [116]. The role of ADP-ribosylation in promoting and in inhibiting viral replication is mentioned in the section below, Figure 3 and Table 1.

#### 3.2.1. Role of ADP Ribosylation in the Inhibition of Viral Replication

PARPs regulate innate immune response at various steps of viral infection. ADP-ribosylation restricts/inhibit viral infection by (i) induction of IFN response. Hosts use MARylating (Mono ADP ribosylating) and non-enzymatic PARPs to modulate IFN response and pro-inflammatory cytokine induction. PARP binds to the RIG-1 receptor and promotes its oligomerization and initialization of the signaling cascade [119]. Using a Venezuelan equine encephalitis virus (VEEV) mutant-based model, Atasheva et al. (2012) identified PARP12, among other PARP proteins, as an essential ISG involved in cellular defense against numerous alphavirus [120].

(ii) Transcriptional silencing of retroviral genome. PARP1 leads to transcriptional silencing of the integrated HIV genome in host cell either by integrating HIV genome in transcriptionally disfavored regions such as the centromere region of chromosomes (Figure 3) or by epigenetic mechanisms, thereby repressing viral infection [121,122]. (iii) Proteasomal degradation of viral proteins and RNA. ZIKV encoded NS1 and NS3 proteins undergo PARP12-mediated PARylation (poly ADP ribosylation). The PAR modification further recruit E3 ubiquitin ligase for ubiquitination. Thus, these two modifications work in synchrony to bring about proteasomal degradation of ZIKV proteins (NS1 and NS3) (Figure 3) [123]. PARP13 is one of the first PARP enzymes for which anti-viral function was identified, and two isoforms ZAP-S (N-terminal tandem zinc-finger motifs that bind RNA) and ZAP-L (N-terminal tandem zinc-finger with C-terminal PARP inactive catalytic domain). PARP13/ZAP-S via N-terminal domain inhibits viral replication by promoting viral RNA degradation by recruiting RNA processing exosomes in HIV-1, Semiliki forest virus [124,125], SINV, and Moloney murine leukemia virus (MLV), alternatively it also binds to 3’-UTR of IFN and causes degradation of IFN (IFNL1, IFNL2, and IFNβ) mRNA [126]. Other PARPs known to inhibit viral replication include PARP5a, PARP7, PARP9, PARP10, PARP12, and PARP13, which inhibit viral replication in one of the ways such as increasing IFNs, ISGs, binding to viral RNA, and preventing its translation [119].

#### 3.2.2. Role of ADP Ribosylation in Promotion of Viral Replication

ADP ribosylation, in addition to inhibiting viral replication, also promotes viral replication. It does so by (i) Inhibition of the IFN response. During IAV replication, PARP1 is localized to the cytosol, where it mediates the IFN alpha receptor (IFNAR) degradation resulting in impaired host anti-viral defense. PARP1 mediated IFNAR degradation depends on PAR-enzymatic activity, although the mechanism is still unclear (Figure 3) [42]. Thus, ADP ribosylation has a potential pro-viral role during influenza virus infection. PARP11 induced during VSV infection causes mono ADP ribosylation of ubiquitin E3 ligase β-transducin repeat-containing protein (β-TrCP), which leads to ubiquitination of IFNAR1 and its degradation. This results in reduced IFN and anti-viral response [127]. To counter host PARPs mediated immune response, several RNA viruses such as alphaviruses, hepatitis E virus, coronavirus [128,129,130] has macrodomain and possess the ability to recognize and reverse the effect of ADP ribosylation. Moreover, the SARS-CoV macrodomain mutant induces a robust pro-inflammatory cytokine response and increases the virus sensitivity to the IFN-1 treatment [130].

(ii) Induction of viral replication. PARP1 is activated upon double-stranded breaks and modifies nuclear histones leading to chromatin decondensation and enhanced access to repair enzymes. The enzyme facilitates HIV-1 integration within the host genome by mediating DNA repair events that follow viral integration [131]. Macrodomain from the RNA virus families bind both mono and poly-ADP-ribose and can hydrolyze and remove mono-ADP-ribose from proteins [132] and promote the replication of viruses [130,133]. CHIKV nsP3 protein macrodomain mutants showed that ADP-ribose binding caused the viral replication initiation, while hydrolase activity is essential in amplifying replication complexes [133]. One other study showed the importance of macrodomain in the HEV (Hepatitis E virus) replication where it was found that macrodomain mutants (N809A and H812L) were nonviable, whereas RNA replication was abrogated in mutants (G816A and G817A) [129]. Another study by Li et al. showed a strong correlation between viral replication and enzymatic activity of the mutant [134]. These findings suggest that macrodomain is not only crucial in replication but also post RNA replication.

## 4. Lipidation

Lipids are significant components of cell membranes and help to maintain cell homeostasis. In addition to their role in the membrane, these are also directly attached to the proteins in the process called protein lipidation. Protein lipidation is the covalent attachment of lipid moieties to proteins [135]. It is an essential post-translation modification involved in processes like membrane localization of proteins, protein–protein interactions, protein stability, enzymatic activity [136,137,138]. Some of the standard types of lipidation examples are palmitoylation, prenylation, and myristoylation [135].

### 4.1. Palmitoylation

Palmitoylation is a reversible type of enzymatic modification in which palmitoyl chain is attached to a cysteine residue called S-palmitoylation involving attachment of 16-carbon palmitoyl group via a thioester linkage) [135]. S-palmitoylation attachment regulates the functions of the proteins by regulating their association with membranes, compartments, trafficking, cellular localization of proteins, and stability [139]. The enzyme responsible for the modification is called Palmitoyltransferase, such as GODZ(, Golgi associated DHHC (Asp-His-His-Cys) zinc finger domain, also known as such as DHHC3 [140]. Initial reports of palmitoylation of envelope glycoproteins of viruses were in SINV and VSV [141,142].

#### 4.1.1. Role of the Palmitoylation in the Inhibition of Viral Replication

The palmitoylation of host proteins plays a vital role in the anti-viral defense strategy. TLRs (Toll-like receptors) recognize nucleic acids of the viruses and triggers the production of type I interferons (IFNs), mainly INFα and IFNβ, and induce an anti-viral reaction in an autocrine and paracrine manner [143]. Interferon-induced transmembrane proteins (IFITMs) are potential viral restriction factors against a wide range of enveloped viruses such as IAV, WNV, DENV, and ZIKV. IFITMs are primarily located in endolysosomal membranes and facilitate the virus’ degradation by blocking their fusion with the membrane (Figure 3) [144,145]. It has also been found that the S-palmitoylation also facilitates the clustering of IFITM3 in the membranes, which suggests that it shows a potential role for its anti-viral activity [146]. Hence, in this way, various host proteins play a crucial role in affecting both the virulence and the host’s immune reactions.

#### 4.1.2. Role of the Palmitoylation in the Promotion of Viral Replication

During the infection, palmitoylation contributes to the virus growth by enhancing virulence, infectivity, cell fusion, viral protein localization, and virion assembly [147,148,149,150]. Palmitoylated viral proteins can be categorized into three categories a) spike proteins, b) viroporins, and c) peripheral membrane protein [151]. Palmitoylation of HCV NS2 is required for NS2-NS3 auto processing and viral assembly, thus, this has an important role in RNA replication (Figure 3) [152]. Similarly, HEV ORF3 is also palmitoylated and plays a role in viral secretion [153]. HIV-1 glycoprotein palmitoylation is required for gp160 incorporation and infectivity [148]. Alphavirus TF (transframe) protein is palmitoylated and is involved in its localization to the plasma membrane and incorporation into a virus [149], and palmitoylated nsP1 is involved in anchoring replication complex to the plasma membrane [154]. The examples mentioned above show that the palmitoylation of viral proteins is crucial for viral replication.

### 4.2. Myristoylation

Myristoylation is the attachment of the myristoyl group (a 14-carbon fatty acid) by N-myristoyltransferases (NMT) to the N-terminal glycine of host and viral proteins. The modification involves protein targeting to membranes, protein-protein interactions, and virus entry [155]. Viral proteins of several viruses such as HIV-1, picornaviruses, Lassa virus, DENV [155,156,157] are known to myristoylated and play an important role in the viral cycle. Inhibition of myristoylation by chemicals such as DDD85646, a pyrazole, and NMT inhibitor, or its knockdown significantly reduces the virus yield indicating an important role of myristoylation in virus maturation and infectivity [157,158].

#### Role of Myristoylation in the Promotion of Viral Replication

Myristoylation of viral proteins like other PTMs helps the viral life cycle processes. HIV-1 matrix protein 31 (MA31) and Nef proteins are myristoylated and have a role in virus assembly and replication (Figure 3). MA31 helps the viral assembly at membrane and myristoylation of MA31 enhances the affinity of protein to membrane, thus helps in assembly. Nef is an early infection protein that increases pathogenicity by downregulating CD4 expression in T cells and helping in virion release [159,160]. Myristoylation of matrix protein Z of Lassa virus, VP4 protein of enterovirus 71 (EV71), and poliovirus polyproteins help in membrane localization and virus assembly, thus in virus replication [156,161,162].

### 4.3. Prenylation

Prenylation is essential for many cellular processes, including signal transduction, cytoskeletal reorganization, and membrane trafficking [163]. It involves the attachment of isoprene lipids (15-carbon (farnesyl) or a 20-carbon (geranylgeranyl)) to the cysteine residue of the proteins. Proteins have CAAX residues at C-terminal (where “C” is cysteine, “A” generally represents an aliphatic amino acid, and the “X residue is serine, methionine, or glutamine) [164]. There are few reports of the role of prenylation during viral infection. In silico analysis predicted prenylation of viral proteins of HIV-1, IAV, HCV, but they are yet to be confirmed.

#### 4.3.1. Role of Prenylation in the Inhibition of Viral Replication

Long isoform of ZAP (Zinc finger anti-viral protein) protein is farnesylated and is targeted to the membrane. The protein inhibits replicating viruses of the *Togaviridae* family such as SINV, Semliki virus (SFV), and RRV by binding to incoming viral RNA (Figure 3) [165].

#### 4.3.2. Role of Prenylation in the Promotion of Viral Replication 

FBL2 (F-box and leucine-rich repeat protein 2), a component of the E3 ubiquitin ligase complex protein, is geranylgeranylated, which interacts with NS5A and plays a role in HCV replication [166]. In addition to host proteins, proteins from several viruses are farnesylated, including the hepatitis delta virus large antigen (lHDAg) [167]. The protein is prenylated at terminal CXXX box and helps in viral assembly [168]. During IAV infection, geranylgeranylation of mitochondrial anti-viral signaling protein (MAVS) inhibits their anti-viral signaling by translocation of Rac1, which is a guanosine triphosphatase to mitochondria-associated endoplasmic reticulum (ER) membranes (MAMs). Rac1 recruitment to MAVS signalosome inhibits the interaction of TRIM31 with MAVS. Rac1 also facilitates the recruitment of Caspase-8, which cleaves MAVS and inhibits MAVS-mediated interferon signaling by cleaving ubiquitinated Receptor-interacting serine/threonine-protein kinase 1 (RIPK1) [169].

## 5. Small Chemical Groups based on Post-Translational Modifications

Small chemical groups such as phosphate (PO_4_^3^^−^), methyl (-CH3), acetyl (-COCH3) from the side chain of amino acids of proteins. These modifications are catalyzed by specific enzymes belonging to families such as kinases/phosphatase, methyltransferase/demethylase, and acetyltransferases/deacetylase. These modifications generally alter the surface charge or act as a binding site for interaction with other proteins [170]. These are well known to play roles in cellular pathways such as replication, metabolic pathways, signaling pathways, and immune pathways [171,172,173,174].

### 5.1. Phosphorylation

Phosphorylation is one of the most conserved types of PTM. The phosphate group is added to amino acids such as serine, threonine (Thr), or tyrosine (Tyr) residues on proteins. It requires adenosine triphosphate (ATP) and is a reversible reaction involving protein kinases and protein phosphatases [171,175]. The phosphorylation of viral and cellular proteins can significantly impact viral infection, replication, and cytotoxicity in a host cell. Eukaryotic cells use these modifications to control the functional repertoire of proteins. Most viral proteins mimic key regulatory factors to usurp this host machinery and promote efficient viral outcomes. Phosphorylation plays a vital role in protein: protein interactions, protein stability, signal transduction, transcription regulation, intracellular localization, cell cycle progression, and apoptosis [176]. Protein phosphorylation is also essential for many intracellular obligate pathogens to establish a productive infection cycle.

#### 5.1.1. Role of Phosphorylation in the Inhibition of Viral Replication

RNA viruses such as VSV, SINV, SeV, and IAV induce interferon response, restricting viral infection [177]. During RNA viral infection, the dsRNAs are recognized by pattern recognition molecules such as RIG-I and dsDNA made during retroviral replication bind cGAS and are converted to cyclic dinucleotides. Both these lead to activation of STING and its translocation to perinuclear vesicles. This leads to the induction of chemokines, type 1 interferon genes, and pro-inflammatory genes via the NF-kB pathway and involves TBK1 (TANK-binding kinase 1) phosphorylation, which in turn phosphorylate IRF3 and STAT6. The cytokines bind to receptors in the neighboring cells, leading to phosphorylation mediated dimerization of STAT protein, which are then translocated to the nucleus and activate the JAK-STAT pathway [178,179]. Influenza NS1 is the canonical antagonist of innate immune responses. It binds to viral RNA, TRIM25, RIG-1, and promotes viral replication and reduces interferon response, but upon phosphorylation during the later stage of infection, its binding to viral RNA, TRIM25, and RIG-1 reduces, thereby negatively affecting viral replication and immune response [180]. Phosphorylation of IAV NS1 and Rubella capsid disrupts protein–RNA interactions and protein-protein interactions [173,181], thereby affecting the viral replication and assembly.

#### 5.1.2. Role of Phosphorylation in the Promotion of Viral Replication

Phosphorylation is involved in signaling pathways and promotes viral growth by (i) increasing the viral replication. Alphaviral nsP3 is heavily phosphorylated at the C-terminal hypervariable domain (HVD), and it shows significant variability in sequence among alphaviruses [182]. In the Semliki Forest virus (SFV), nsP3 C-terminal tail is involved in the activation of the phosphatidylinositol-3-kinase (PI3K)-Akt-mammalian target of Rapamycin (mTOR) pathway as well as replication complex formation. The hyper phosphorylated tail of SFV leads to AKT activation, resulting in efficient internalization of replication complexes in SFV, but not the Chikungunya virus (CHIKV) [183]. Other examples include Ebola VP35, a viral RNA-dependent RNA polymerase cofactor, and its activity is phosphorylation- dependent; thus, phosphorylation has a positive role in Ebola viral replication [184].

(ii) Increasing the viral budding. The phosphorylation of matrix protein Z of the Lassa virus at Y97and S98 of PPXY late domain (Pro-Pro-X-Tyr domain, a motif involved in virus release) regulates the virus release by regulating the budding process. The proteins associate with membrane and helps’ in recruitment of NP to membrane where viral assembly and release takes place [185]. (iii) Inhibition of immune pathways. Flaviviral non-structural protein 5 (NS5) inhibits interferon-mediated anti-viral response by targeting host JAK-STAT protein, and through inhibition of anti-viral signaling pathways. DENV and other flaviviral such as ZIKV, and Yellow fever virus NS5 bind to STAT2, preventing its phosphorylation, which is required to form complex with JAK and further translocated to nucleus for anti-viral responsive gene expression [186] and target it to proteasomal degradation [187,188]. Nucleocapsid (N) protein binds to B23 (a nucleolar phosphoprotein and substrate for CDK2/cyclinE, involved in centrosome replication and regulates cell cycle), STAT, and prevents their phosphorylation. This negatively affects the cell cycle and interferon response [189,190]. SARS-CoV nucleoprotein (N) phosphorylation may also affect its nucleocytoplasmic shuttling by interaction with the host adapter protein [191].

### 5.2. Methylation

Methylation of viral RNA is a well-studied viral growth regulation mechanism [192], whereas the post-translational role of methylation during RNA viral infection is not well studied. The virus utilizes several approaches for its full replication and immune evasion strategy by co-opting the transcriptional and translational regulation of infected cells to produce new viral particles [193]. S-adenosyl methionine (SAM) acts as a methyl group donor, and transfer is catalyzed by methyltransferases.

#### 5.2.1. Role of Methylation in the Inhibition of Viral Growth

**Figure 4:**
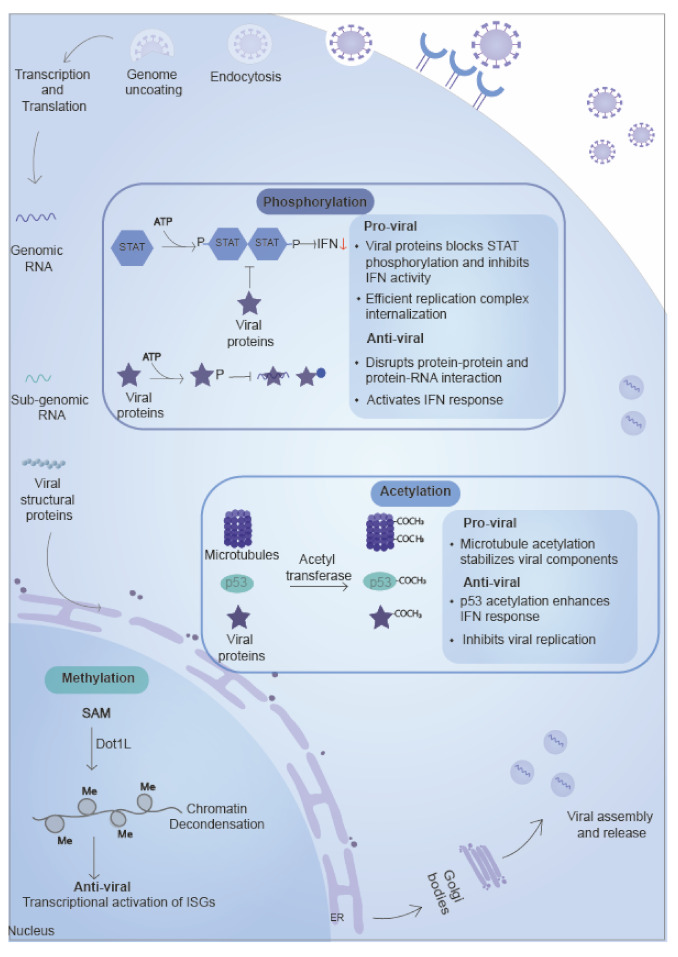
Small molecules/chemical group based PTMs. Small chemical groups such as phosphate, methyl, acetyl, and ionic groups are added to proteins by specific enzymes. Phosphorylation involves kinases which transfer phosphate group and play a role in protein-protein interaction, thereby affecting protein localization and signaling pathways. Methylation of host and viral proteins involved methyl group transfer from substrate protein by the help of methyltransferases. Methylation plays a role in modulating gene expression by regulating chromatin condensation, regulating both host gene expression and transcription of integrated viral genomes. Acetylation of histone proteins regulate gene expression and microtubule organization and affect viral replication.

Retroviruses such as HIV integrate into the host genome and be in the latent phase by regulating host histone methylation. SEC (Super Elongation Complex) promotes H3K27 acetylation at HIV-1 LTR (long terminal repeat), which leads to transcription elongation, and the same complex also recruits CARM1, a methyltransferase, and causes H3R26 (histone 3 arginine 26) methylation leading to attenuation [194]. HIV Tat protein promotes the expression of MeCP2 (methyl CpG binding protein 2) and Ezh2 (Enhancer of zeste homolog 2, a histone-lysine N-methyltransferase enzyme), which induces Tri-methylation of H3K27 leading to attenuation [195]. In addition to histones, viral proteins are also methylated, which controls virus latency. Methylation of Tat by SETDB1 inhibits transactivation of HIV LTR [196], whereas demethylation by lysine-specific demethylase 1 (LSD1/KDM1) activates HIV transcription [197]. During influenza infection, LSD1 is induced by IFNα and demethylate and activates IFITM3 leading to infection suppression (Figure 4) [198].

### 5.3. Acetylation

Acetylation is one of the most common PTMs present in histone protein. It is involved in the global regulation of gene expression during cell differentiation and development by histone protein modification [199]. Acetylation also affects viruses at different stages of cycles like entry, replication, fusion, transport, and release [200]. The modification is most prevalent on lysine amino acid and is catalyzed by acetyltransferases belonging to the family of GNAT (Gcn5-related N-acetyltransferases), MYST (histone acetyltransferases), and p300/CBP (E1A binding protein p300/CREB-binding protein) localized in the nucleus, cytoplasm as well as in mitochondria [199].

#### 5.3.1. Role of Acetylation in the Inhibition of Viral Replication

Acetylation and deacetylation of viral proteins and human immune pathway proteins affect their activity leading to inhibition of viral replication, e.g., acetyltransferases (PCAF and GCN5) mediated acetylation of influenza nucleoprotein (NP) protein near to RNA binding groove (K31 and K90) inhibit the viral replication (Figure 4) [201]. In addition to directly acetylating/deacetylating viral proteins, the host also modifies its proteins to enhance the anti-viral effect as seen in the p53 protein, which is acetylated during infection and leads to transactivation of pro-apoptotic and IFN-stimulated genes (Figure 4) [202]. Deacetylases are essential in regulating gene expression due to their role in chromatin remodeling. Histone deacetylase (HDAC) removes the acetyl group from histone molecules and inhibit gene expression. NF-kB p50 mediated recruitment of HDAC1 to HIV LTR lead to repression of transcription due to impairment of RNA Pol II recruitment [203]. Other HDAC, HDAC6, causes deacetylation of RIG-I at C-terminal enhances RNA sensing, or prevents the trafficking of IAV virus to membrane or RNA Pol subunit PA leading to proteasome degradation [200,204,205], thereby inhibiting the viral replication.

#### 5.3.2. Role of Acetylation in the Promotion of Viral Replication

Acetylation of influenza viral protein such as NP (K77, K113, and K229) and NS1 (K108) play a positive role in their replication and growth via increase the protein’s activity and IFN antagonism [206,207]. Acetylation of viral proteins such as HIV Tat promotes transcription of the integrated genome by recruiting the positive transcription elongation factor (pTEFb) complex at LTR promoter [197]. In contrast to inhibiting viral replication, HDAC6, in association with rabies matrix protein (M), promotes viral replication by depolymerizing tubulin [208]. Another HDAC, HDAC4 during Sendai virus infection, binds to TBK1/IKKε and prevents phosphorylation of IRF3, which is needed for the production of type I IFN [209]. Microtubules are structural components and are involved in providing structural support as well as cellular transport. Viral infections are found to induce acetylation of microtubules and stabilize viral compartments in the cytoplasm of cells in the case of noroviruses, rotavirus, and reovirus, and promotes viral replication (Figure 4) [210,211]. HDAC inhibition using chemical inhibitors in some viruses such as Japanese encephalitis virus (JEV) and RSV results in the inhibition of viruses, indicating the role of acetylation in viral replication [212,213].

## 6. Conclusions

Post-translational modifications of proteins are vital to the cellular processes of both hosts as well as viral proteins. These help to maintain homeostasis by regulating their solubility, stability, interaction with partners, and degradation. The PTMs help the host combat viral infection by activating the immune system and degrading viral proteins and playing a positive role for viruses where they help in viral assembly, enzymatic activity of viral protein, and interferon response inhibition. The dominance of the immune system’s activation to control viral replication and control over the immune system decides the outcome of the disease. Recently a few studies have shown that targeting host proteins involved in post-translational modifications such as kinases can be effective in restricting viral replication. Additionally, targeting the proteins involved in the immune response reduces the complications related to immune activation in viral infection such as SARS-CoV-2, IAV, alphavirus, and flavivirus, and could be a way to reduce the damage caused by the host to itself. More future studies about the mechanism and components involved in post-translational modifications in various viruses can reveal more targets for the treatment of infections.

## Figures and Tables

**Table 1:** PTMs and their role during viral infection.

Modification	Enzyme	Target Protein	Impact	References
Ubiquitination				
Anti-viral	E3 Ligase	NS3 (DENV) Integrase (HIV) and Core (HCV)	Ubiquitin mediated proteasomal degradation	[17,19]
	USP11	Nucleoprotein (NP) (Influenza)	Deubiquitination of mono-ubiquitinated NP which inhibits its enzymatic activity	[29]
	USP49	APOBEC3G (Host A3G)	Deubiquitination and stabilization of A3G causing inhibition of viral replication via C to U leading to TGA to TAA termination	[30,31]
Pro-viral	Cul4A, DDB1	STAT1, STAT2, and STAT3 (Host)	Proteasomal degradation of STAT proteins which inhibits type I IFN response	[32]
	CRL, TRIM6	PB2 (IAV), VP35 (Ebola)	Enhances polymerase action and thus viral replication	[6,25]
	CNOT4	NP (IAV)	Promotes replication and assembly	[26]
**ISGylation**				
Anti-viral		NS3 and NS5(DENV)	Suppresses virion release	[33]
		NS1 (IAV)	Reduces binding to viral RNA and importin-alpha which is needed for its nuclear transport	[34]
Pro-viral		Crimean-Congo haemorrhagic fever virus (CCHFV, family *Nairoviridae*)	Viral OTU protease mediated deISGylation suppressing interferon responses	[35]
	HERC5	NS5A(HCV)	Promotes viral replication via recruitment of cyclophilin A	[36]
**SUMOylation**				
Anti-viral		TRIM28	SUMOylation of TRIM28 inhibits immunostimulatory dsRNAs generation by suppression of ERV (endogenous retroviral element). dsRNA play role in activation of IFN response via binding to RIG-1 during infection with influenza virus	[37]
		Enterovirus 71 (EV71) 3C protease	SUMOylation promote 3C degradation which inhibit viral replication and promote apoptosis	[38]
Pro-viral		Enterovirus 71 RNA-dependent RNA polymerase, 3D Pol	SUMOylation enhances stability and activity of 3D protein, thus enhances the viral replication	[39]
		CDK9	SUMOylation of CDK9 inhibits its kinase activity thereby inhibit interaction of Cyclin T and RNA Pol II CTD phosphorylation causing HIV latency	[40]
**ADP Ribosylation**				
Anti-viral	PARP 13	PB2, PA (IAV)	ADP ribosylation of the PB2, PA proteins promotes recognition by E3 ubiquitin ligase and subsequent degradation	[41]
Pro-viral	PARP1	Type I Interferon Receptor (IFNAR1)	ADP ribosylation by PARP1promotes proteasomal degradation of IFNAR1 during IAV infection	[42]
